# First Report of *Pichia bruneiensis* in a Spontaneous Sugarcane Juice Fermentation: A Case Study from an Artisanal Distillery in the Ecuadorian Amazon

**DOI:** 10.3390/bioengineering13020254

**Published:** 2026-02-22

**Authors:** Marcos David Landívar Valverde, Mayra Vanessa Chiriboga Ruilova, Estela Guardado Yordi, Amaury Pérez Martínez

**Affiliations:** Facultad de Ciencias de la Vida, Universidad Estatal Amazónica, Puyo 160104, Ecuador; mv.chiribogar@uea.edu.ec (M.V.C.R.); e.guardadoy@uea.edu.ec (E.G.Y.)

**Keywords:** isolation, yeast, *Pichia*, native microbiota, sugarcane spirit

## Abstract

Spontaneous fermentation of sugarcane juice for the production of artisanal sugarcane spirits in the Ecuadorian Amazon is driven by native microbial communities; however, the yeast diversity involved in this process remains poorly characterized. In this descriptive case study, sugarcane juice samples were collected from a single artisanal distillery at three fermentation stages (0, 48, and 96 h). Yeasts were isolated using selective culture techniques, yielding three distinct morphotypes (Y01, Y02, and Y03). A progressive reduction in morphological diversity was observed during fermentation, with isolate Y01 persisting at the final stage (96 h). Differences in crystal violet staining indicated variability in cellular metabolic activity among the isolates under the conditions tested. Biochemical characterization using the API 20C AUX system, combined with molecular identification based on ITS rDNA sequencing, was performed for isolate Y01, which showed ≥99% sequence identity with *Pichia bruneiensis*. To the best of our knowledge, this study represents the first report of *P. bruneiensis* associated with spontaneous sugarcane juice fermentation in an artisanal distillery from the Ecuadorian Amazon. These findings provide a descriptive baseline on yeast biodiversity in this traditional fermentation system and support future studies aimed at the functional characterization of native yeasts involved in artisanal sugarcane spirit production.

## 1. Introduction

Artisanal sugarcane spirits, locally known as aguardiente, are traditionally produced in the Ecuadorian Amazon, particularly in Pastaza Province [1]. This region comprises approximately 4500 ha of sugarcane cultivation, representing about 55% of the total area planted in the Ecuadorian Amazon [2]. Within this context, nearly twenty small-scale distilleries are dedicated to artisanal spirit production [3]. These production systems rely primarily on empirical knowledge transmitted across generations and are deeply embedded in local cultural and socioeconomic practices.

From a technical standpoint, the production of artisanal sugarcane spirits is characterized by the spontaneous fermentation of freshly extracted sugarcane juice, followed by distillation. During this process, sugars naturally present in the juice are converted into ethanol through microbial activity. Fermentation typically proceeds for approximately five days under ambient conditions, without the addition of commercial starter cultures or controlled inoculation [4]. Consequently, the process is driven by native microbial communities associated with the raw material, the production environment, and local handling practices. A similar production system has also been reported for chachaça in Brazil [5].

Spontaneous fermentations involve complex and dynamic microbial consortia, in which yeasts play a central role in sugar metabolism and ethanol production [6,7]. Previous studies on traditional fermented beverages have demonstrated that native yeasts contribute to microbial diversity and influence fermentation outcomes [8,9]. However, the composition and temporal dynamics of yeast communities involved in spontaneous sugarcane juice fermentation remain insufficiently characterized, particularly in underexplored regions such as the Ecuadorian Amazon [10]. While genera such as *Pichia* have been reported in spontaneous fermentations of plant-derived substrates, their occurrence and persistence in artisanal sugarcane juice fermentations have not been systematically documented.

In contrast to traditional practices, modern alcohol production increasingly relies on highly domesticated yeast strains—mainly *Saccharomyces cerevisiae*, *S. pastorianus*, and *S. bayanus*—due to their predictable fermentation performance and operational reliability [11,12,13,14]. Although these strains offer clear technological advantages, their widespread and exclusive use has been associated with reduced microbial diversity and increased homogenization of fermentation systems [15,16]. Documenting native microbial communities in traditional fermentations remains important for understanding the biodiversity associated with culturally significant production systems. In recent years, increasing attention has been paid to the role of non-Saccharomyces yeasts in spontaneous fermentations due to their contribution to microbial diversity and metabolic complexity [17].

In the Ecuadorian Amazon, previous research on artisanal distilleries has primarily focused on technical, environmental, and process-related aspects, including waste management, energy efficiency, and environmental impact reduction [4,18]. These studies have identified challenges such as raw material losses, energy inefficiencies, and limited operational control. However, the spontaneous fermentation stage—which is central to alcohol production and the preservation of traditional practices—has received limited attention from a microbiological perspective. As a result, there is a lack of information regarding the yeasts involved in this process.

Therefore, the objective of the present study was to isolate and identify yeasts associated with the spontaneous fermentation of sugarcane juice in a single artisanal distillery in the Ecuadorian Amazon. By providing a descriptive characterization of yeast morphotypes and molecular identification of dominant isolates, this work aims to contribute baseline information on native yeast biodiversity in this traditional fermentation system and to support future studies focused on the functional characterization of indigenous yeasts.

## 2. Materials and Methods

### 2.1. Materials

The sugarcane juice samples were collected using sterile 500 mL flasks at an artisanal distillery located in the rural settlement of Teniente Hugo Ortiz, Pastaza Province, Ecuador. Culture media were prepared using potato dextrose agar (PDA; Difco^®^, Detroit, MI, USA) supplemented with chloramphenicol (Ecuaquímica^®^, Quito, Ecuador) to inhibit bacterial growth. Sterile distilled water was used for serial dilutions, and gentian violet solution (Novachem^®^, Quito, Ecuador) was used for microscopic staining.

Biochemical characterization of yeast isolates was carried out using the API 20C AUX commercial system (bioMérieux^®^, Marcy-l’Étoile, France). Molecular identification was performed by Sanger sequencing of the internal transcribed spacer (ITS) region of ribosomal DNA. PCR amplification was conducted using primers ITS1 (5′-TCCGTAGGTGAACCTGCGG-3′) and ITS4 (5′-TCCTCCGCTTATTGATATGC-3′), synthesized by Macrogen^®^, Seoul, South Korea.

### 2.2. Sample Collection

Sugarcane juice samples were collected at three stages of the spontaneous artisanal fermentation process: immediately after the fresh juice entered the fermentation tank (0 h), after 48 h, and after 96 h of fermentation (Table 1). Sampling at multiple time points was conducted to obtain a descriptive overview of yeast occurrence during fermentation, following the general approach reported by [19].

As a negative control, a fresh sugarcane juice sample was collected and sterilized at 121 °C for 15 min. All samples were transported under refrigeration (4 °C) to the Biology Laboratory at Universidad Estatal Amazónica and processed immediately upon arrival to minimize microbial alterations, as recommended by [19].

### 2.3. Isolation of Yeasts

Serial dilutions of each sample were prepared in sterile distilled water up to 10^−5^, following the procedure described by [20,21]. Aliquots of 300 µL from each dilution were spread in triplicate onto PDA plates supplemented with 25 mg/L chloramphenicol, according to yeast isolation protocols reported by [22]. Plates were incubated at 25 °C for 72 h.

#### 2.3.1. Colony Selection and Pure Cultures

After incubation, colonies displaying morphological characteristics consistent with yeasts—such as shape, color, margin, and elevation—were selected. Presumptive yeast colonies were re-streaked onto fresh PDA plates supplemented with chloramphenicol to obtain pure cultures, following the method described by [23].

Microscopic examination was performed after simple staining with gentian violet. Observations were conducted under oil immersion at 1000× magnification to confirm yeast-like cellular morphology, as described by [24]. This staining procedure was used solely for morphological observation and not as a quantitative or definitive indicator of cell viability or fermentative performance.

#### 2.3.2. Identification of Isolates

Preliminary phenotypic characterization of yeast isolates was carried out using the API 20C AUX system (bioMérieux^®^), which provides carbohydrate assimilation profiles for yeast differentiation [22]. Biochemical profiling was used as a complementary tool and not as a definitive method for species identification.

Molecular identification was performed exclusively for isolate Y01, which persisted at the final stage of fermentation. Genomic DNA was extracted, and the ITS region of ribosomal DNA was amplified by PCR using primers ITS1 and ITS4, following the protocol described by [25]. Amplification products were purified and sequenced using the Sanger method [26]. Resulting sequences were compared with reference sequences deposited in the GenBank^®^ database using the Basic Local Alignment Search Tool (BLASTn; NCBI BLAST version 2.15.0+) provided by the National Center for Biotechnology Information (NCBI).

## 3. Results

### 3.1. Sample Collection, Isolation and Colony Selection and Pure Cultures of Yeasts

Yeast-like colonies were detected in all non-sterilized sugarcane juice samples (SS1, SS2, and SS3), indicating the presence of culturable yeasts throughout the spontaneous fermentation process. No yeast growth was observed in the sterilized control sample (CS1). A qualitative reduction in colony morphological diversity was observed at the final fermentation stage.

Based on macroscopic characteristics, three distinct yeast colony morphotypes were identified and designated Y01, Y02, and Y03. Differentiation was based on colony color, surface texture, border morphology, and elevation (Table 2). All three morphotypes were recovered from samples collected at the initial (0 h) and intermediate (48 h) fermentation stages (SS1 and SS2). In contrast, only morphotype Y01 was detected in samples collected at 96 h (SS3).

Microscopic observation following simple staining revealed differences in cell morphology among the isolates. Isolate Y01 consisted predominantly of ovoid cells exhibiting multilateral budding, whereas isolates Y02 and Y03 displayed apiculate or ellipsoidal cell shapes, with occasional pseudomycelium observed in Y03 (Table 2).

**Figure 1:**
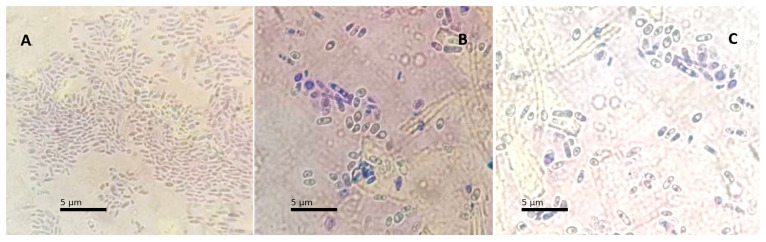
Comparison of crystal violet staining between isolate Y01 (**A**) and isolates Y02 (**B**) and Y03 (**C**).

Differences in crystal violet staining patterns were also observed among the isolates. Isolate Y01 exhibited low dye retention, while isolates Y02 and Y03 showed higher levels of staining (Figure 1). Crystal violet staining was used solely as a qualitative indicator of differential dye retention among isolates and not as a quantitative measure of viability, stress tolerance, or fermentative performance.

### 3.2. Identification of Isolates by API 20C AUX and Taxonomic Identification with ITS Sequencing

Isolate Y01 was selected for further identification due to its persistence across all sampling points and its detection at the final fermentation stage (96 h). Phenotypic characterization using the API 20C AUX system revealed a carbohydrate assimilation profile consistent with yeasts belonging to the genus *Pichia*. Isolate Y01 assimilated glucose, fructose, sucrose, trehalose, and cellobiose, and showed variable assimilation of maltose and sorbitol (Table 3). Biochemical profiling was used as a supportive phenotypic tool and not as a definitive method for species-level identification.

**Figure 2:**
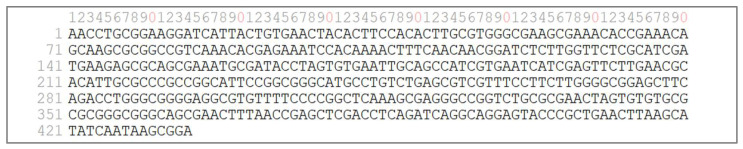
Consensus sequence of isolate Y01 (*P. bruneiensis*).

Molecular identification was performed by sequencing the internal transcribed spacer (ITS) region of ribosomal DNA. Sequencing using primer ITS1 produced a read of 423 bp, while sequencing with primer ITS4 generated a read of 425 bp. The overlapping regions of both reads were aligned to generate a consensus sequence of 434 bp corresponding to the ITS1–5.8S–ITS2 region (Figure 2).

The consensus sequence was deposited in FASTA format and compared against reference sequences in the GenBank database using the BLAST algorithm. Sequence comparison showed ≥99% identity with reference sequences annotated as *Pichia bruneiensis*. This molecular result supports the assignment of isolate Y01 to *P. bruneiensis* based on ITS rDNA analysis.

BLAST analysis of the ITS rDNA consensus sequence showed ≥99% sequence identity and 100% query coverage with *Pichia bruneiensis* reference sequences deposited in the GenBank database. The sequence obtained in this study was deposited in GenBank under accession number PX741098.

## 4. Discussion

The present study provides a descriptive characterization of culturable yeasts associated with the spontaneous fermentation of sugarcane juice in a single artisanal distillery located in the Ecuadorian Amazon. Although the experimental scope was limited to one production system and one fermentation batch, the results offer initial insight into the yeast diversity present during this traditional process and contribute baseline microbiological information for an underexplored fermentation context.

A qualitative reduction in colony morphological diversity was observed as fermentation progressed, with three morphotypes detected during the early and intermediate stages and only one morphotype (Y01) recovered at the final sampling point (96 h). Similar qualitative shifts in yeast occurrence have been reported in spontaneous fermentations of plant-derived substrates, where changes in physicochemical conditions over time may influence the culturability of different yeast populations [5,9]. However, given the absence of quantitative microbial counts and fermentation parameters in the present study, no conclusions can be drawn regarding microbial succession, dominance, or competitive fitness among isolates.

Isolate Y01 was selected for molecular identification due to its persistence across all sampling points. ITS rDNA sequencing assigned this isolate to *Pichia bruneiensis* with ≥99% sequence identity and full query coverage relative to reference sequences deposited in GenBank. While the ITS region is widely used for yeast identification, it is recognized that ITS-based resolution within the genus *Pichia* may be limited for closely related taxa [25]. Nevertheless, the high sequence identity obtained, together with phenotypic consistency, supports the taxonomic assignment of isolate Y01 as *P. bruneiensis*. Future studies incorporating multilocus sequence analysis or whole-genome sequencing would provide higher-resolution taxonomic confirmation.

The carbohydrate assimilation profile obtained using the API 20C AUX system was consistent with profiles reported for species within the genus *Pichia* [22]. As previously noted, biochemical profiling alone is not sufficient for definitive species identification but remains a useful complementary approach when interpreted alongside molecular data. In the present study, API 20C AUX results were therefore used to support, but not replace, sequence-based identification.

Differences in crystal violet staining patterns were observed among the yeast isolates, with lower dye retention in isolate Y01 compared to isolates Y02 and Y03. Crystal violet staining was used exclusively as a qualitative microscopic tool and does not provide direct information on cell viability, stress tolerance, or fermentative performance [24]. Consequently, no functional interpretation of staining behavior is proposed. The observed differences may reflect variations in cell wall properties among morphotypes, which could be explored in future studies using quantitative physiological assays.

The detection of *P. bruneiensis* in spontaneous sugarcane juice fermentation represents, to the best of our knowledge, the first report of this species in this specific fermentation system. Previous studies on sugarcane-based fermentations, particularly those conducted in Brazil, have primarily reported the presence of *Saccharomyces* species and other non-*Saccharomyces* yeasts [12,15,27], but *P. bruneiensis* has not been explicitly documented. This finding expands the known ecological distribution of the species and highlights the microbial diversity associated with traditional fermentations in the Ecuadorian Amazon.

Although species of the genus *Pichia* have been reported in various fermented foods and beverages [28,29,30], the present study does not evaluate functional traits such as ethanol tolerance, aroma compound production, or fermentative performance. Any potential technological or biotechnological applications of isolate Y01 therefore remain hypothetical and should be addressed through targeted physiological, metabolic, and safety assessments. In this context, the current work should be regarded as exploratory, providing a foundation for future research rather than evidence for immediate application.

From a broader perspective, documenting native yeasts in spontaneous fermentations contributes to the understanding of microbial biodiversity associated with culturally significant food and beverage production systems [6,15,31]. In regions such as the Ecuadorian Amazon, where artisanal practices are closely linked to local identity and traditional knowledge, such baseline microbiological information is essential for future studies aiming to balance product quality, cultural preservation, and sustainability.

## 5. Conclusions

This study provides a descriptive characterization of culturable yeasts associated with the spontaneous fermentation of sugarcane juice in a single artisanal distillery located in the Ecuadorian Amazon. Three yeast morphotypes were isolated during the fermentation process, with one isolate (Y01) persisting until the final fermentation stage.

Molecular identification based on ITS rDNA sequencing assigned isolate Y01 to *Pichia bruneiensis* with ≥99% sequence identity and full query coverage relative to reference sequences deposited in GenBank. To the best of our knowledge, this represents the first report of *P. bruneiensis* associated with spontaneous sugarcane juice fermentation in this context.

Given the descriptive scope and methodological limitations of the present work, no functional or technological properties can be inferred for the isolated yeasts. Consequently, the findings should be interpreted as baseline microbiological information that contributes to the documentation of native yeast biodiversity in traditional fermentation systems of the Ecuadorian Amazon.

Future studies incorporating quantitative fermentation parameters, broader molecular identification, and targeted physiological and safety assessments will be necessary to evaluate the functional roles and potential applications of indigenous yeasts identified in this system.

## Figures and Tables

**Table 1:** Coding of the sugarcane juice samples.

Code	Description
CS1	Fresh and sterilized cane juice (negative control)
SS1	Sample collected at the beginning of fermentation (Time 0 h)
SS2	Sample collected at 48 h of fermentation (Time +48 h)
SS3	Sample collected at 96 h of fermentation (Time +96 h)

**Table 2:** Morphological characterization of the yeast isolates.

Colony ID	Macroscopic Morphology	Microscopic Morphology	Violet Staining	SS1 (0 h)	SS2 (48 h)	SS3 (96 h)
Y01	Creamy-white, slightly wrinkled surface, regular border	Ovoid cells, multilateral budding, no pseudomycelium	Low	+	+	+
Y02	Opaque white, rough surface, irregular border	Apiculate cells, polar budding, no pseudomycelium	High	+	+	-
Y03	Bright white, moist, well-defined circular border	Ellipsoidal cells, some with pseudomycelium	High	+	+	-

Notes: Affinity to crystal violet staining was classified as high (≥80% of cells stained intensely), medium (40–79%), or low (<40%) by microscopic observation (1000×). The presence of growth at each time was recorded as “+” when typical colony formation was observed on solid medium after seeding and incubation at 28 °C for 48 h, and as “-” when no growth was evident.

**Table 3:** Carbohydrate assimilation results from the API 20C AUX assay.

No.	Substrate (Carbohydrate)	Isolate Y01	*Pichia* spp. *	*Saccharomyces cerevisiae* *
1	Glycerol	+	+	+
2	Erythritol	-	-	-
3	D-Xylose	+	+	-
4	L-Arabinose	-	-	-
5	Ribose	+	±	-
6	D-Glucose	+	+	+
7	D-Fructose	+	+	+
8	Galactose	+	+	+
9	Sucrose	+	+	+
10	Maltose	±	±	+
11	Lactose	-	-	-
12	Trehalose	+	+	+
13	Cellobiose	+	+	-
14	Inositol	-	-	-
15	Sorbitol	+	+	-
16	Methyl-α-D-glucopyranoside	+	+	+
17	N-Acetyl-glucosamine	±	±	-
18	Arbutin	±	+	-
19	Salicin	±	+	-
20	D-Glucuronate	-	-	-

Notes: “+” means positive growth/assimilation, “±” means weak/limited assimilation, and “-” means it does not assimilate substrate. * Reference taken from [22].

## Data Availability

The dataset generated during the current study, consisting of the ITS rDNA sequence of *Pichia bruneiensis* isolate Y01, has been deposited in the GenBank database (NCBI) under accession number PX741098. The sequence will be publicly available upon release by GenBank or at the time of publication. During peer review, access to the sequence can be provided by the corresponding author upon reasonable request. All the data generated in the research is in the manuscript.
